# The Views of Patients with Isolated Rapid Eye Movement Sleep Behavior Disorder on Risk Disclosure

**DOI:** 10.1002/mds.29403

**Published:** 2023-04-12

**Authors:** Laura Pérez‐Carbonell, Cristina Simonet, Harneek Chohan, Aneet Gill, Guy Leschziner, Anette Schrag, Alastair J. Noyce

**Affiliations:** ^1^ Sleep Disorders Centre, Guy's and St Thomas' NHS Foundation Trust London United Kingdom; ^2^ Preventive Neurology Unit, Wolfson Institute of Population Health Queen Mary University of London London United Kingdom; ^3^ Department of Clinical and Movement Neuroscience UCL Institute of Neurology London United Kingdom

**Keywords:** autonomy, risk disclosure, Parkinson's disease, isolated REM sleep behavior disorder

## Abstract

**Background:**

Isolated rapid eye movement sleep behavior disorder (iRBD) is associated with an increased risk of Parkinson's disease and other synucleinopathies. There is no consensus about disclosure of this risk to patients with iRBD.

**Objective:**

The objective of our study was to assess the experiences of risk disclosure in a group of patients with iRBD and their views on what, when, and how this should be done.

**Methods:**

A survey was administered to patients with iRBD to explore their experiences and views on risk disclosure.

**Results:**

Thirty‐one patients with iRBD (28 males; mean age, 70 [SD 8.7] years; mean disease duration, 8.7 [SD 6.4] years) were included. A third reported they had not been informed about the link between iRBD and other conditions by clinicians at diagnosis, but 90% would have liked to have received prognostic information, and 60% indicated that this should happen at the point that iRBD was diagnosed. Most participants wanted this information to come from the clinician diagnosing and treating iRBD (90.3%). Almost three‐quarters (72.2%) had searched for this information online.

**Conclusions:**

Patients with iRBD mostly wished to have received information regarding the potential implications of iRBD when the diagnosis was made. © 2023 The Authors. *Movement Disorders* published by Wiley Periodicals LLC on behalf of International Parkinson and Movement Disorder Society.

## Introduction

Isolated rapid eye movement (REM) sleep behavior disorder (iRBD) is characterized by dream‐enactment behaviors during REM sleep and documented REM sleep without atonia during polysomnography.^1^ A high proportion of patients with iRBD eventually develop a neurodegenerative condition, such as Parkinson's disease (PD) or dementia with Lewy bodies.^2^ A recent large multicenter study demonstrated a cumulative phenoconversion rate of 73.5% at 12‐year follow‐up and an overall conversion rate of 6.3% per year.^3^

Despite this very high risk, there is ongoing debate around the information that should be disclosed to patients with iRBD and how this should be done.^4^, ^5^, ^6^, ^7^, ^8^, ^9^, ^10^, ^11^ The implications of risk disclosure have been studied in other clinical scenarios.^12^, ^13^, ^14^, ^15^, ^16^, ^17^, ^18^ However, there has been little published on disclosing risk to individuals in the prodromal phase of PD and related disorders.^19^, ^20^, ^21^, ^22^

The aim of our study was to assess the experiences of risk disclosure in a group of patients with iRBD and their views on what, when, and how this should be done.

## Patients and Methods

In this exploratory study, a structured questionnaire was designed by the study team to understand the preferences of patients with iRBD for receiving information about the link between iRBD and neurodegenerative conditions (Supporting Information Box S1).

### Survey

The first part of the questionnaire included demographic and clinical information. The second part of the survey comprised nine multiple‐choice questions focused on two main aspects: (1) their experiences regarding receiving information about the association of iRBD with neurodegenerative conditions at the time of diagnosis; and (2) what, when, and how they would have liked to have been informed. A final open‐answer question allowed them to provide advice to clinicians on iRBD counseling.

### Participants

Participants with iRBD had previously been recruited to the PREDICT‐PD study^23^ and were initially diagnosed with iRBD at the Sleep Disorders Centre, Guy's Hospital in London. All patients had a diagnosis of iRBD confirmed by video polysomnography. Patients with secondary RBD (not related with an α‐synucleinopathy) or a concurrent neurodegenerative condition were excluded.

Written informed consent was obtained from all participants. Ethics approval was granted by the Queen Square Research Ethics Committee (09/H0716/48). All methods were performed in accordance with the relevant guidelines and regulations.

## Results

A total of 31 participants with iRBD completed the questionnaire (Table 1). Most patients (90%) were male, with mean age of 70 (SD 8.7) years. The mean reported disease duration of confirmed iRBD diagnosis was 8.7 (SD 6.4) years.

**TABLE 1:** Demographic and clinical information

Demographic and clinical characteristics	iRBD Participants (n = 31)
Age, y (SD)	70 (8.7)
Sex (male:female)	28:3
Years of iRBD diagnosis (SD)	8.7 (6.4)
Psychiatric symptoms, n (%)	Depression: 2 (6.4%)
	Anxiety: 4 (12.9%)
	Both: 3 (9.7%)
	No problems: 22 (71%)
Memory difficulties, n (%)	Mild: 14 (45.2%)
	Moderate: 2 (6.4%)
	No problems: 15 (48.4%)

### Questions 1–4: Participants’ Experience Regarding Risk Disclosure

More than a third of participants (11/31, 35.5%) reported they had not received any information about the link of iRBD with neurodegenerative conditions at the time of diagnosis. Of those, half searched for further information online (n = 6/11, 54.5%).

Among those who received information concerning their risk (n = 18), eight participants recalled being asked whether they wanted to know more beforehand, two were not asked, and eight did not recall. More than two‐thirds of those who received information from their clinician (13/18, 72.2%) had also searched online.

Two participants could not recall whether risk disclosure information was shared with them; one of them searched online.

### Questions 5–7: What Would You Have Liked to Be Told?

Most participants (28/31, 90.3%) had wanted to receive information regarding the potential future implications of iRBD, and they cited reasons around autonomy and contributions to research as the main drivers. Two people would have not wanted to know because it was their right and they felt that there was nothing that they could do about the future. One participant did not respond.

### Question 8: From Whom Would You Have Liked to Be Told?

Twenty‐eight (90.3%) participants would have liked to receive information about their risk from the physician diagnosing and treating their iRBD. Of those choosing an alternative source, two people preferred their primary care doctor, and one patient cited information leaflets and scientific websites. A quarter of participants endorsed the use of information leaflets and being directed to websites alongside discussions with their specialist (8/31, 25.8%).

### Question 9: When Would You Have Liked to Be Told?

The preferred time to receive information about risk was at the time of iRBD diagnosis (19/31, 61.3%). Two participants preferred being told only when parkinsonism or dementia symptoms emerged, and two would have liked to be told if treatments to reduce or stop the risk to develop PD and related conditions became available. Eight participants (25.8%) did not report any preferences about the timing.

### Question 10: Advice to Clinicians When Discussing iRBD Implications

Participants advocated for honesty and good communication (including with primary care clinicians). As well as the free text included in the questionnaire, three participants suggested disclosing iRBD implications using a stepwise process, starting with broad terms and moving on to more detailed information based on patients' needs.

## Discussion

Our study demonstrated that more than a third of this group of patients with iRBD did not receive information at diagnosis about the links between their sleep condition and risks to develop a neurodegenerative disease. In less than half of instances, participants recalled that their clinician had ascertained their preferences about disclosure first. Most patients indicated that they would like to have been told about the potential association between iRBD and neurodegenerative diseases to plan for the future and participate in research. These results are in line with the findings of Gossard and colleagues^22^ in a recently published study using a similar approach. Most of their respondents felt that knowing about the risk was important for future planning and expressed an interest in receiving information about neuroprotective therapies.^22^

The need for a consensus to approach risk disclosure in iRBD has been previously stressed.^10^ The findings from our study highlight that practices, even in a single specialist center, remain heterogeneous. However, principles of autonomy, beneficence, nonmaleficence, and justice, considered within medical ethics, should always prevail and be applied when risk disclosure to a patient with iRBD is being considered.^24^

In this context, ensuring the autonomy of patients involves exploring whether they are willing to know or not know about the risk of developing a neurodegenerative disorder,^10^, ^11^ moving away from a default position of medical paternalism or deciding for the patient.^25^ A recent study involving 101 patients with PD reported that less than half would have liked to know about their risk of PD before the diagnosis, although this proportion increased to 85% if the disclosure had included recommendations about lifestyle changes (eg, exercise, diet) that might have altered the course of the disease.^19^ In a survey‐based study, less than a third of clinicians had asked patients' preferences about receiving risk information, albeit they felt counseling helped maintain trust in the patient–doctor relationship.^21^ Patients' and clinicians' characteristics appear to influence prognostic discussions. A study including data of patients with iRBD or REM sleep without atonia reported several determinants for prognostic counseling, including clinicians' sex (male) and specialty (neurology) and patients' age (>60 years) and primary complaint (iRBD suspicion vs. this being an incidental/secondary finding).^20^

Receiving information about the risk of developing a neurodegenerative disorder allows patients to plan for the future and is important to relatives who may step into a “pre‐caregiver” role.^26^, ^27^ As shown in a recent study, almost 70% of RBD experts considered that a potential benefit of risk disclosure was future planning.^21^ In addition, being aware of one's risk would allow participation in clinical trials when neuroprotective therapies become available.^5^, ^28^ This is timely because in 2023 both drug and nondrug (eg, exercise‐based) intervention studies will launch for patients with iRBD with the goal of reducing the probability of phenoconversion. Risk disclosure brings the possibility to advise a change in health behaviors^14^, ^18^, ^19^, ^29^ and can lead to early symptomatic treatment with the goal to improve patients' quality of life.^30^ In contrast, risk disclosure can have a psychological impact on individuals,^15^, ^16^, ^31^ especially in the absence of proven disease‐modifying therapies.^32^, ^33^ Importantly, one study reported that a quarter of patients with PD would endorse psychological support after risk disclosure during a prodromal phase of their condition.^19^ The potential impact on patients' and relatives' mental health also appears to be a concern for most clinicians involved in iRBD counseling.^21^

The participants who were willing to know felt that being aware of the evidence would allow informed decision‐making for the future and would bring the possibility to participate in clinical trials. Those who would have not wanted to have their risk disclosed reported that it was their right as patients to not know, and that they would rather not have the information if there was nothing that could be currently done about what might happen in the future. These findings stress the fact that an individualized approach is very much needed when disclosing risk to a patient with iRBD.^22^ Less than half of the participants in our study were asked about their preferences before the risk disclosure conversation took place.

Most participants in our study preferred to have their risk disclosure discussed with the doctor diagnosing and treating their iRBD and at the time of the iRBD diagnosis. Importantly, almost two thirds of patients searched for information about iRBD online, which is also reported after diagnosis of other neurological disorders.^34^ Furthermore, within those having had a risk discussion with their doctor, the majority searched for further information online. As per our findings, facilitating access to scientifically sound materials (eg, patient information leaflets and scientific or patients' associations websites) for those patients willing to receive information should be included in our routine care of patients with iRBD.

**FIG 1:**
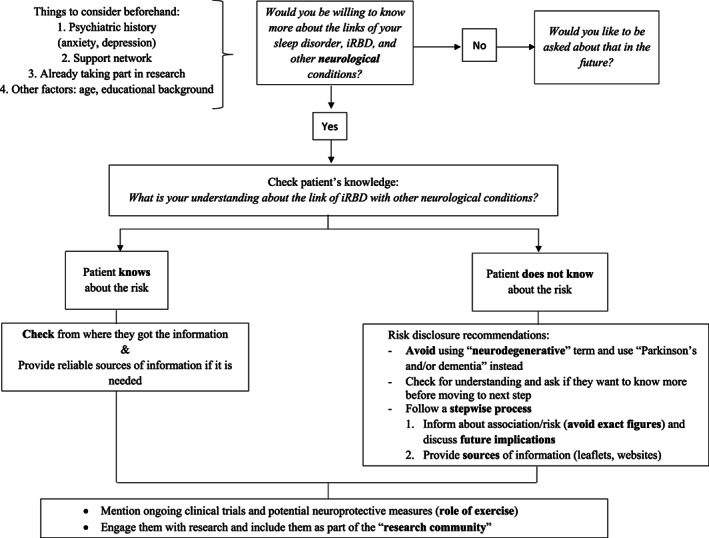
Suggested stepwise approach for risk disclosure in isolated rapid eye movement sleep behavior disorder (iRBD). This is based on the clinical experience of the authors and takes into account the patients' answers given to the open question in our questionnaire and the patients' views informally expressed while the questionnaire was undertaken.

Overall, patients' opinions and preferences should be considered and respected as part of enabling the principle of autonomy and to become aware of the potential impact that risk disclosure may have for each individual.^6^ A suggested stepwise approach to the risk disclosure discussion is included in Fig. 1. Initiating the conversation with open questions and without overly detailed information at the outset appears to be appropriate (Fig. 1).

Our study has some limitations. There is the potential for selection bias in the participants included in this study. These individuals were interested and already collaborating in iRBD research, and all of them were aware of the risks of neurodegenerative conditions linked to iRBD. This may have an impact on their views regarding risk disclosure because their potential involvement in clinical trials with neuroprotective therapies might also be more appealing for this subset of patients. Arguably, these patients may, however, also be those who are most aware of the negative impact of risk disclosure. Other limitations are that the sample size was small and there was a high proportion of males included (ratio 9:1), although it is recognized that RBD tends to affect males more than females. Furthermore, the participants were all recruited from a single sleep center in the United Kingdom, which may limit the generalizability of the findings. Studies comprising a larger and multicenter sample, alongside the inclusion of clinicians' views, and exploring current best practice related to the discussion of disease‐modifying approaches and lifestyle changes are important avenues for future research.

## Conclusions

Most people with iRBD would have liked to have information regarding the potential future implications of this condition, preferably at the time of iRBD diagnosis and from their specialist, but combined with other information sources (leaflets/scientific websites). A personalized approach, carefully eliciting patients’ wish for disclosure, preserving patients' autonomy, and providing appropriate information at the time of diagnosis should be adopted by clinicians managing patients with iRBD.

## Author Roles

L.P.‐C.: design, execution, writing, and editing of final version of the manuscript. C.S.: design, execution, writing, and editing of final version of the manuscript. H.C.: analysis and editing of final version of the manuscript. A.G.: editing of final version of the manuscript. G.L.: editing of final version of the manuscript. A.S.: editing of final version of the manuscript. A.J.N.: design, writing, and editing of final version of the manuscript.

## Financial Disclosures of All Authors (for the Preceding 12 Months)

L.P.‐C.: none. C.S.: none. H.C.: none. A.G.: none. G.L.: speaker fees for Takeda, consultancy fees for Eisai, and institutional income from Bioproject and Jazz. A.S. received research funding or support from University College London, National Institute of Health, National Institute for Health Research ULCH Biomedical Research Centre, the International Parkinson and Movement Disorder Society, the European Commission and Parkinson's UK; honoraria for consultancy from Biogen and AbbVie; and University College London Royalties from Oxford University Press. A.J.N. reports grants from Parkinson's UK, Barts Charity, Cure Parkinson's, National Institute for Health and Care Research, Innovate UK, Virginia Keiley benefaction, Solvemed, the Medical College of Saint Bartholomew's Hospital Trust, Alchemab, Aligning Science Across Parkinson's Global Parkinson's Genetics Program (ASAP‐GP2), and The Michael J. Fox Foundation. A.J.N. reports consultancy and personal fees from AstraZeneca, AbbVie, Profile, Roche, Biogen, UCB, Bial, Charco Neurotech, uMedeor, Alchemab, Sosei Heptares, and Britannia, outside the submitted work.

## Supporting information


**Data S1.** Supporting Information.

## Data Availability

The data that support the findings of this study are available from the corresponding author upon reasonable request.
