# Supplementary material for: The Views of Patients with Isolated Rapid Eye Movement Sleep Behavior Disorder on Risk Disclosure
Source: Mov Disord. 2023 Apr 12;38(6):1089–93. doi: 10.1002/mds.29403 (PMC10947281; doi:10.1002/mds.29403)
Supplement: Supplementary file 1 [file MDS-38-1089-s001.docx]

Box 1. Questionnaire developed.

Part 1

Next we would like to ask you some questions about what you were t[illegible] time of your diagnosis and how you were told.

1. Did your doctor give you information about the link of iRBD with other co[illegible] including developing a neurodegenerative condition (such as Parkinson's dis[illegible] future?

Yes, No, I don't know/remember

2. If yes, did your doctor ask what you wanted to know prior to giving this in[illegible]

Yes, No, I don't know/remember

3. Were you provided with patient information leaflets, websites or other [illegible] information to know more about the links between iRBD and neurod[illegible] conditions?

Yes, No, I don't know/remember

4. Apart from the information provided (if this was given), did you [illegible] information about iRBD on the internet?

Yes, No, I don't know/remember

Part 2

Now we would like to ask you some questions about whether your
would have been different to what happened (i.e. what, when and how have liked to be told?)

5. Did you want to have information regarding the potential future impl having iRBD at that time?

Yes, No, I don't know

If the answer is "No", please go to question 6.

If the answer is "Yes", please go to questions 7, 8 and 9.

6. What were the reasons why you did NOT want to know about the poten implications of iRBD at that time? (mark all that apply)

It is my right to NOT know, as I am the patient

If there is nothing that I can do about what may happen in the future, I'd rather

I would have liked to know, but at a later date - please give detail

I would have liked to know, but be told differently - please give detail

Other reasons [free text]

7. What were the reasons that you did WANT to know about the poten implications of iRBD? (mark all that apply)

It is my right to know, as I am the patient

I wanted to have all the information so that I could make informed decisions f

Even if there is nothing that I can do about what may happen in the future, interested in participating in research related to iRBD

Other reasons [free text]

8. From where would you have liked to receive the information about th future implications of iRBD? (mark all that apply)

By the doctor diagnosing and treating my iRBD

By my GP

With information in a patient information leaflet

With information online from scientific and patients' associations websites

9. Did you have a preference regarding when to be told about the poten implications of iRBD?

No.

Yes, from the moment I received the diagnosis of iRBD.

Yes, when my doctor detected initial signs of parkinsonism.

Yes, but later in life, if treatments to reduce or stop the risk to develop Park related conditions became available.

[illegible]
